# Exposure to herbivore-induced plant volatiles primes JA-dependent gossypol defenses in cotton

**DOI:** 10.1371/journal.ppat.1014338

**Published:** 2026-06-11

**Authors:** Chuanyu Zhu, Chengcheng Yao, Runxiao Liu, Tao Sun, Changsong Zou, Qingsong Liu, Sergio Rasmann, Ted C. J. Turlings, Yunhe Li

**Affiliations:** 1 State Key Laboratory of Crop Stress Adaptation and Improvement, State Key Laboratory of Cotton Bio-breeding and Integrated Utilization, School of Life Sciences, College of Agriculture, Henan University, Kaifeng, China; 2 State Key Laboratory for Biology of Plant Diseases and Insect Pests, Institute of Plant Protection, Chinese Academy of Agricultural Sciences, Beijing, China; 3 Laboratory of Fundamental and Applied Research in Chemical Ecology, Laboratory of Functional Ecology, University of Neuchȃtel, Neuchȃtel, Switzerland; INRAE, FRANCE

## Abstract

Herbivore-induced plant volatiles (HIPVs) are known to prime neighboring plants for enhanced defense, but the molecular basis for this phenomenon remains poorly understood, particularly in cotton. Here, we demonstrate for cotton plants that exposure to volatiles induced by the cotton bollworm (*Helicoverpa armigera* Hübner; CBW), enhances resistance against subsequent CBW attack, as evidenced in both laboratory and semi-field trials. While HIPV exposure alone did not elicit direct defense activation, it primed the jasmonic acid (JA) signaling pathway, leading to accelerated induction of JA biosynthesis genes and elevated JA accumulation upon herbivory. That this primed resistance is JA-dependent, was confirmed by treating HIPV-exposed plants with JA biosynthesis inhibitors, which completely abolished the priming effects. We further found that HIPV-primed plants exhibited significantly higher accumulation of the key defensive metabolite gossypol following larval feeding. The role of gossypol-reliant defenses was confirmed by using a glandless cotton mutant (*gl*_*2*_*gl*_*2*_*gl*_*3*_*gl*_*3*_) deficient in gossypol and related terpenoid aldehydes. The combined results reveal that CBW-induced volatiles prime anti-herbivore resistance in cotton by potentiating the JA signaling pathway, which in turn enhances gossypol biosynthesis upon actual herbivore attack. This new insight into the physio-ecological mechanisms underlying airborne defense priming in cotton also highlights its potential application in sustainable pest management.

## Author summary

Plants emit chemical scents to alert each other to danger, and the phenomenon is referred to as plant-plant communication. While these airborne signals are known to either directly activate plant defenses or prime neighboring plants against subsequent herbivore attack, the underlying molecular mechanisms in cotton remain poorly understood. Using the cotton bollworm (*Helicoverpa armigera*) as a model, we revealed that cotton volatiles induced by this pest do not directly trigger defenses in neighboring cotton plants. Instead, these volatiles prime the plants to initiate a more rapid and robust defensive response upon subsequent attack by the conspecific pest. Our results further demonstrated that cotton volatiles act as airborne signals to first activate the JA signaling pathway, then boost the production of defensive metabolites such as gossypol, and ultimately enhance the plant’s resistance to cotton bollworms. These findings provide new insight into the physio-ecological significance of information exchange mediated by herbivore-induced plant volatiles in cotton and lay a foundation for developing eco-friendly pest management strategies using plants’ innate chemical defenses.

## Introduction

Despite their sessile nature, plants have evolved a sophisticated and diverse array of defense strategies to cope with abiotic and biotic stresses, particularly insect herbivory [1–5]. Herbivores in turn develop counter-adaptations that modulate, suppress, or circumvent plant defenses, resulting in a continual evolutionary arms race [6,7]. Among the most ecologically widespread defense mechanisms are plant volatiles, particularly herbivore- or oviposition-induced plant volatiles (HIPVs/OIPVs), which mediate interactions not only between plants and insects but also among conspecific plants [8–11].

Originally characterized as cues that attract natural enemies of herbivores, HIPVs are now known to play broader roles in plant defense orchestration [12–14]. Increasing evidence indicates that undamaged plants can perceive HIPVs emitted by their herbivore-attacked neighbors and adjust their own defenses accordingly [15,16]. Such volatile-mediated plant–plant signaling can either directly induce defense gene expression or, more commonly, establish a primed physiological state in which defenses are activated more rapidly and robustly after subsequent attack [17–20]. HIPV-mediated priming has been documented in multiple plant species, including maize, rice, tea, and sweet potato, where exposure to volatiles such as green leaf volatiles, indole, or DMNT leads to enhanced phytohormone signaling, elevated metabolite accumulation, or increased herbivore resistance [21–26]. However, volatile cues can also suppress plant defenses under specific ecological contexts, demonstrating that the outcome of HIPV signaling is context- and species-dependent [27,28].

Cotton, a globally important fiber crop, faces persistent threats from a wide array of insect pests throughout its life cycle [29,30]. A key component of the cotton defense system is the presence of specialized pigment glands, which constitutively accumulate a variety of defensive compounds such as terpenes, flavonoids, and tannins [31]. These glands are critical for resistance to herbivory, as glandless cotton varieties exhibit significantly heightened susceptibility to phytophagous insects [32–34]. Among these defensive metabolites, gossypol, a terpenoid aldehyde known for its toxicity to mammals and its adverse effects on the lepidopteran larvae is of particular importance. It is abundantly sequestered in pigment glands, and its biosynthesis can be further induced upon pest attack [35,36]. This inducible accumulation of gossypol is primarily regulated by the jasmonic acid (JA) signaling pathway [37]. It has been well documented that HIPVs as airborne signals can activate the JA signaling pathway in plants [26], promote the biosynthesis of downstream defensive metabolites such as gossypol, and consequently enhance plant resistance against insect pests. A recent study by Grandi et al. (2024) [38] reported that “old” HIPVs, those released 24–48 hours after *Spodoptera littoralis* caterpillar feeding, triggered direct defense responses in neighboring cotton plants, increasing JA levels and upregulating defense-related genes. In contrast, “new” HIPVs emitted within the first 24 hours after damage did not elicit such responses. However, the study focused solely on direct induction and did not assess whether these HIPVs contribute to defense priming linked to JA-dependent gossypol accumulation.

The cotton bollworm (*Helicoverpa armigera* Hübner; CBW), inflicts severe economic losses on cotton, by feeding directly on reproductive structures, thereby reducing yield and compromising fiber quality [39]. Although early observations suggested that cotton plants may respond to volatiles emitted by herbivore-infested neighbors [38,40,41], the mechanistic basis of this phenomenon remains largely unresolved. In particular, it is unknown whether HIPVs released by CBW-infested plants can prime neighboring cotton plants for enhanced defenses, nor whether such priming involves the induction of key metabolites such as gossypol. Addressing these gaps is critical for understanding how cotton integrates volatile cues into its defensive strategy and how these processes might be exploited for sustainable pest control.

Here, we hypothesize that exposure to CBW-induced enhances resistance in neighboring cotton plants through a gossypol-mediated priming mechanism. To test this, we first evaluated CBW larval performance on receiver plants exposed to volatiles from CBW-infested or uninfested emitters under laboratory and semi-field conditions. We then investigated the molecular and biochemical foundations of HIPV-mediated priming, with a particular focus on defense signaling pathways and gossypol accumulation in receiver plants. Finally, by employing a gene-edited cotton line with reduced gossypol production, we assessed the causal role of gossypol in HIPV-induced resistance. Together, our findings provide new insight into the physio-ecological significance of HIPV-based information exchange in cotton and offer a foundation for developing pest management strategies that leverage plants’ innate chemical defenses.

## Results

### Exposure to CBW-induced volatiles enhances cotton resistance against conspecifics

**Fig 1:**
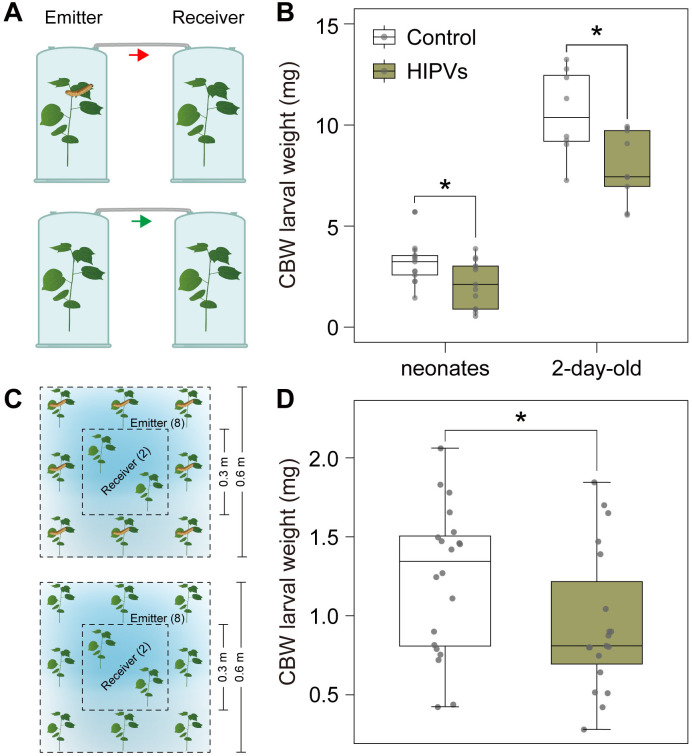
Weight of cotton bollworm (CBW) larvae feeding on receiver plants pre-exposed to different volatiles in the laboratory and semi-field trail. **(A)** Schematic diagram illustrating the exposure of receiver plants exposed to volatiles from emitter plants infested with 3^rd^-instar CBW larvae. Control plants were exposed to volatiles from uninfested plants under the same conditions. **(B)** After a 48 h exposure to volatiles from either uninfested plants (Control) or from plants infested for 24 h with two 3^rd^-instar CBW larvae (HIPVs), individual receiver plants were infested with four uniformly sized CBW larvae and allowed to feed for 72 **h.** The mean larval weight per plant was then recorded (neonates: *n* = 13; 2-day-old larvae: *n* = 8-9; Student’s *t*-tests). **(C)** Schematic of the semi-field setup in which two receiver plants were exposed to volatiles from eight emitter plants either infested with 3^rd^-instar CBW larvae or left uninfested. **(D)** Receiver plants were initially exposed for 24 h to volatiles from either uninfested or CBW-infested plants and then infested with eight uniformly sized neonate CBW larvae. After 72 h of feeding, the mean larval weight per plant was measured (*n* = 19-20; linear mixed models followed by Bonferroni-adjusted pairwise comparisons). Throughout the outdoor bioassays, receivers remained adjacent to the emitters until trial completion. Asterisks indicate significant differences between treatments (*, *P* < 0.05).

Laboratory and semi-field bioassays were carried out to evaluate the resistance of cotton plants exposed to volatiles from uninfested or CBW-infested plants against CBW. Across two consecutive indoor experiments (**Fig 1A**), CBW caterpillars feeding for 72 h on plants pre-exposed to CBW-induced volatiles exhibited significantly lower body weight than those feeding on control plants exposed to volatiles from uninfested emitters (**Fig 1B***, t* = 2.317, df = 24, *P* = 0.029 for neonates, and *t* = 2.786, df = 15, *P* = 0.014 for 2-day-old caterpillars). Semi-field trials (**Fig 1C**) corroborated these results, showing similarly reduced larval performance on HIPV-exposed plants (**Fig 1D**; *F*_1,35_ = 5.996, *P* = 0.020).

### HIPVs-exposure enhances JA signaling gene expression and JA accumulation in herbivore-attacked receiver plants

**Fig 2:**
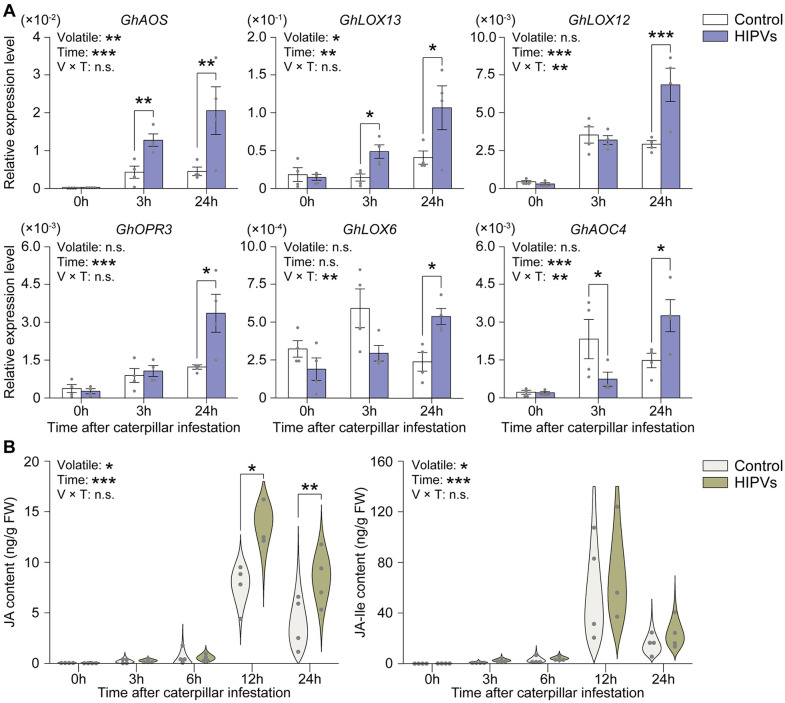
Jasmonic acid (JA)-related genes expression and phytohormone contents in receiver plants. Expression profiles of six JA signaling-related genes **(A)** and contents of JA and JA-isoleucine (JA-Ile) **(B)** in receiving plants that had been exposed for 48 h to volatiles from uninfested or CBW-infested plants following CBW caterpillar infestation at different time points. Gene abbreviations: *GhAOS*, *G. hirsutum* allene oxide synthase; *GhLOX13*, *G. hirsutum* lipoxygenase 13; *GhLOX12*, *G. hirsutum* lipoxygenase 12; *GhOPR3*, *G. hirsutum* 12-oxophytodienoate reductase 3; *GhLOX6*, *G. hirsutum* lipoxygenase 6; *GhAOC4*, *G. hirsutum* allene oxide cyclase 4. Bars represent mean ± SE. Asterisks indicate significant differences between treatments (*n* = 4; two-way ANOVA followed by pairwise comparisons through Bonferroni adjustment; *, *P* < 0.05; **, *P* < 0.01; ***, *P* < 0.001).

Because JA signaling plays a central role in cotton defense against CBW [42], we examined the expression of eight key JA biosynthetic genes. HIPV exposure alone did not alter transcript levels (all *P* > 0.050). However, following subsequent CBW feeding, HIPV-exposed plants showed distinct transcriptional responses (**Fig 2A**). After 3 h of herbivory, *GhAOS* and *GhLOX13* were significantly upregulated (*F*_1,18_ = 8.732, *P* = 0.008; *F*_1,18_ = 6.574, *P* = 0.020, respectively), whereas *GhAOC4* was downregulated (*F*_1,18_ = 7.623, *P* = 0.013). After 24 h, six genes—*GhAOS*, *GhLOX13*, *GhLOX12*, *GhOPR3*, *GhLOX6*, and *GhAOC4*—showed significant upregulation (*F*_1,18_ = 11.029, *P* = 0.004; *F*_1,18_ = 4.798, *P* = 0.042; *F*_1,18_ = 19.419, *P* < 0.001; *F*_1,18_ = 6.575, *P* = 0.020; *F*_1,18_ = 6.129, *P* = 0.023; *F*_1,18_ = 5.162, *P* = 0.036, respectively). The expression of *GhLOX1* and *GhOPR2* remained unchanged (*P >* 0.050, **S1 Fig**).

JA levels mirrored these patterns (**Fig 2B**). HIPV exposure alone did not affect JA content (*F*_1,29_ = 0.001, *P* = 0.978), but following herbivory, JA accumulation increased significantly at both 12 h (*F*_1,29_ = 4.599, *P* = 0.041) and 24 h (*F*_1,29_ = 7.528, *P* = 0.010). JA-Ile level also showed an upward trend following herbivory, although this increase was not statistically significant.

### JA signaling is required for HIPVs-mediated resistance in cotton

**Fig 3:**
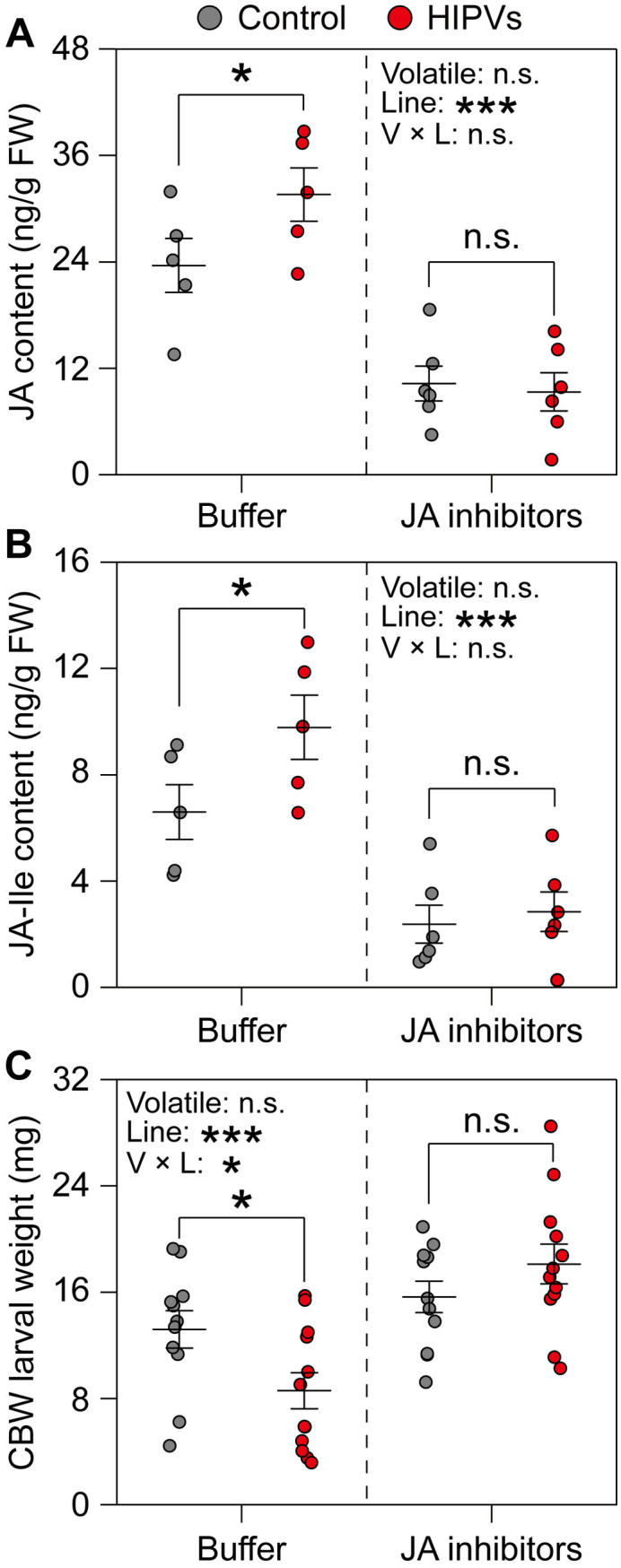
JA signaling is pivotal for regulating CBW resistance in receiver plants. Contents of JA **(A),** JA-Ile **(B)** in receiver plants pre-exposed for 48 h to volatiles from either uninfested or CBW-infested cotton plants, and treated with either buffer (*n* = 5) or JA synthesis inhibitors (SHAM and DIECA) (*n* = 6) prior to a 24 h CBW infestation. **(C)** After exposure to differentially treated volatiles and buffer or inhibitors treatments, individual receiver plant was infested with four uniformly sized 2-day-old CBW larvae. Following 72 h of feeding, the mean larval weight per plant was measured (*n* = 11-12). Asterisks indicate significant differences between treatments (two-way ANOVA followed by pairwise comparisons through Bonferroni adjustment; *, *P* < 0.05; ***, *P* < 0.001).

To further verify the functional role of JA signaling pathway in the HIPV-mediated cotton resistance, a bioassay was conducted including a plant treatment with JA biosynthesis inhibitors (200 μM SHAM + 100 μM DIECA). HIPV-exposed plants accumulated higher levels of JA and JA-Ile after CBW feeding than controls (**Fig 3A, 3B**; JA: *F*_1,18_ = 4.606, *P* = 0.046; JA-Ile: *F*_1,18_ = 5.550, *P* = 0.030). Yet, this priming effect was abolished when JA biosynthesis was chemically inhibited, with no differences between HIPV-exposed and control plants in JA or JA-Ile levels (**Fig 3A, 3B**; JA: *F*_1,18_ = 0.076, *P* = 0.786; JA-Ile: *F*_1,18_ = 0.142, *P* = 0.711).

Consistently, CBW larvae gained significantly less weight when feeding on HIPV-exposed plants than on controls (**Fig 3C**; *F*_1,42_ = 5.698, *P* = 0.022). However, this growth reduction was no longer observed when JA biosynthesis was inhibited (**Fig 3C**; *F*_1,42_ = 1.637, *P* = 0.208). This could be attributed to the fact that under JA inhibition, HIPV exposure failed to induce the JA-dependent accumulation of gossypol in cotton (**S2 Fig**; *F*_1,12_ = 1.239, *P* = 0.287). Together, these results demonstrate that JA signaling is indispensable for the HIPV-primed resistance in cotton.

### HIPVsexposure enhances gossypol accumulation in herbivore-attacked receiver plants

**Fig 4:**
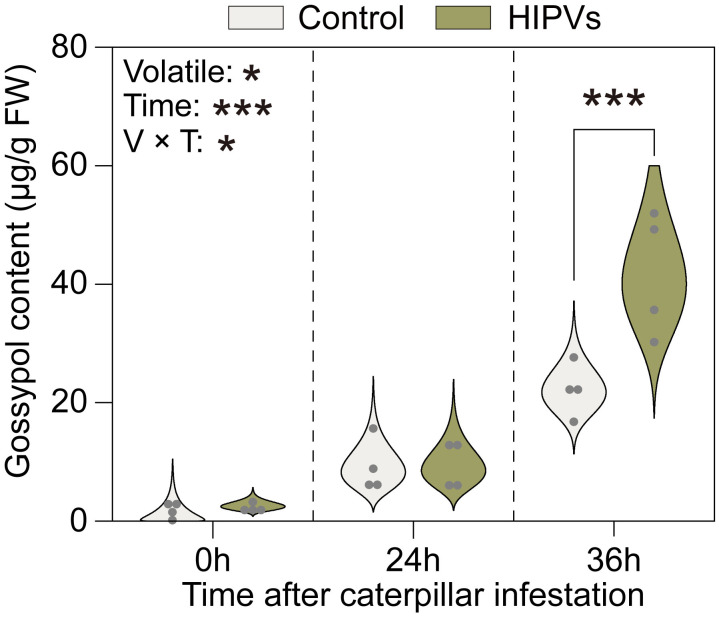
Gossypol content in receiver plants. Content of gossypol in receiving plants that had been pre-exposed for 48 h to volatiles from uninfested or CBW-infested plants following CBW caterpillar infestation at different time points. Asterisks indicate significant differences between treatments (*n* = 4; two-way ANOVA followed by pairwise comparisons through Bonferroni adjustment; *, *P* < 0.05; ***, *P* < 0.001).

Gossypol is a known broad-spectrum insect-resistant compound in cotton, and it is regulated by JA-signaling pathway [43], we therefore further measured gossypol levels in receiver plants pre-exposed to volatiles from differentially treated plants. Pre-exposure to CBW-induced volatiles alone did not significantly alter gossypol content in the absence of herbivory, nor after 24 h of subsequent CBW infestation (**Fig 4**). However, after 36 h of CBW feeding, HIPV-exposed plants exhibited significantly higher gossypol accumulation than controls (**Fig 4**; *F*_1,18_ = 15.606, *P* < 0.001).

### HIPVs-primed resistance in cotton depends on gossypol accumulation

**Fig 5:**
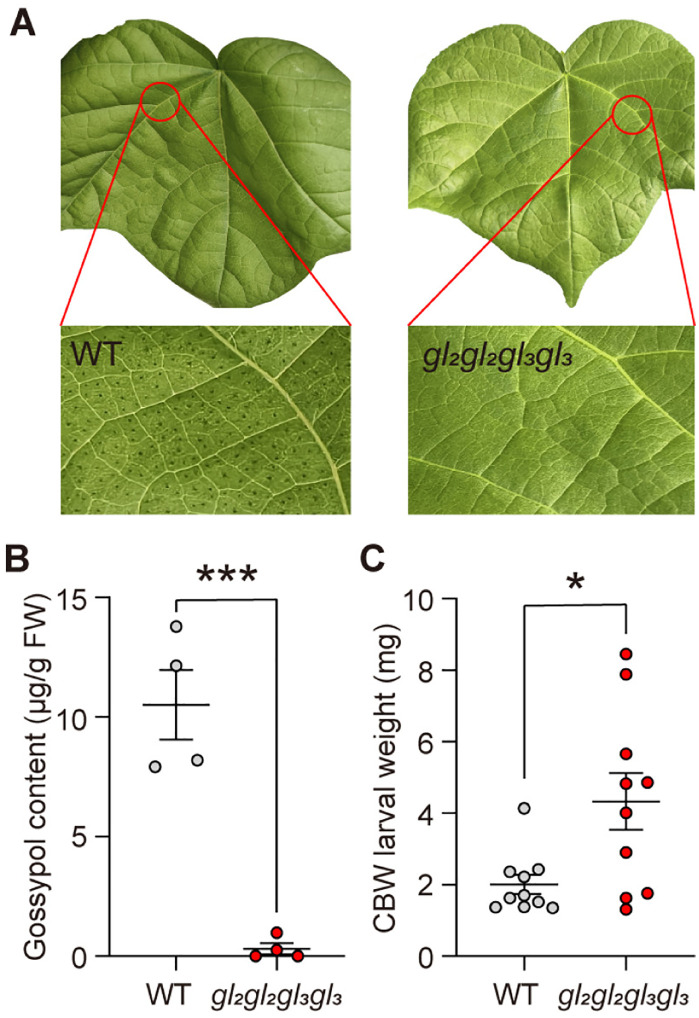
The *gl*_*2*_*gl*_*2*_*gl*_*3*_*gl*_*3*_ mutant reduces gossypol production and CBW resistance in cotton. **(A)** Representative images of WT and *gl*_*2*_*gl*_*2*_*gl*_*3*_*gl*_*3*_ mutant cotton plants. **(B)** Content of gossypol in the leaves of WT plants and *gl*_*2*_*gl*_*2*_*gl*_*3*_*gl*_*3*_ mutants (*n* = 4). **(C)** Weight of CBW larvae after 72 h of feeding on WT or *gl*_*2*_*gl*_*2*_*gl*_*3*_*gl*_*3*_ mutants (*n* = 10). Asterisks indicate significant differences between treatments (Student’s *t*-tests; *, *P* < 0.05; ***, *P* < 0.001).

The homozygous recessive mutant with the genotype *gl*_*2*_*gl*_*2*_*gl*_*3*_*gl*_*3*_ mutant lacks pigment glands (**Fig 5A**), resulting in markedly reduced gossypol production (**Fig 5B**; *t* = 6.915, df = 6, *P* < 0.001) and enhanced larval growth compared to wild type (WT) (**Fig 5C**; *t* = 2.767, df = 18, *P* = 0.013).

**Fig 6:**
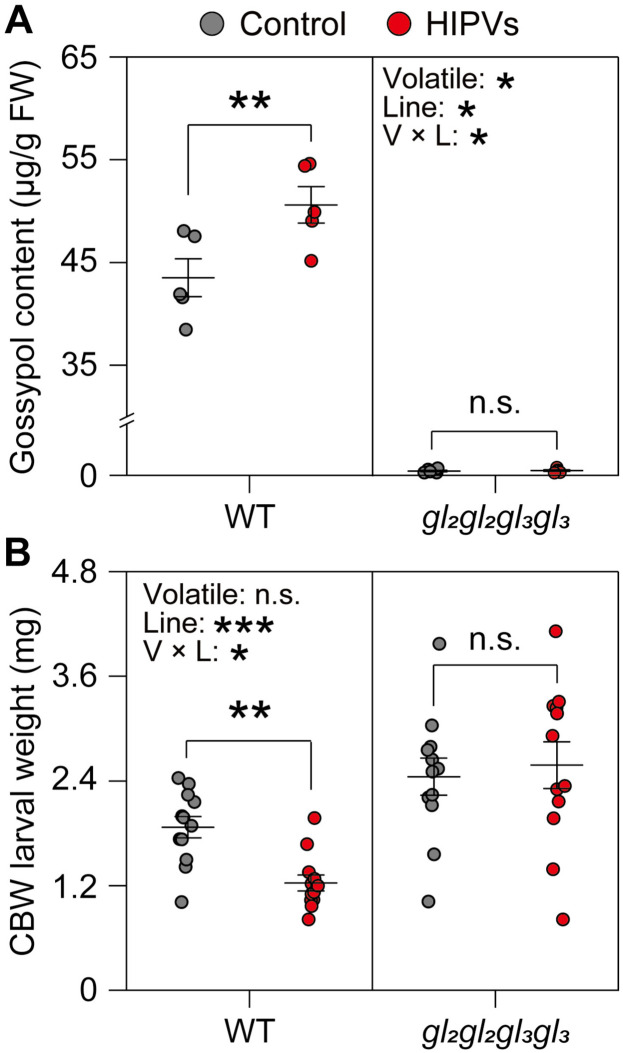
Gossypol biosynthesis in glands is required for regulating CBW resistance in receiver plants. **(A)** Content of gossypol in WT or *gl*_*2*_*gl*_*2*_*gl*_*3*_*gl*_*3*_ mutants that were pre-exposed for 48 h to volatiles from either uninfested WT plants or WT plants that were infested with CBW larvae for 24 h, followed by subsequent CBW infestation for 72 h (*n* = 5). **(B)** Weight of CBW larvae after 72 h of feeding on WT or *gl*_*2*_*gl*_*2*_*gl*_*3*_*gl*_*3*_ mutants that had been exposed to differentially treated cotton volatiles (*n* = 12). Asterisks indicate significant differences between treatments (two-way ANOVA followed by pairwise comparisons through Bonferroni adjustment; *, *P* < 0.05; **, *P* < 0.01; ***, *P* < 0.001).

To investigate whether HIPV-primed resistance relies on gossypol biosynthesis, we assessed the effects of HIPV exposure on both WT and *gl*_*2*_*gl*_*2*_*gl*_*3*_*gl*_*3*_ mutant plants. In WT plants, pre-exposure to HIPVs significantly enhanced gossypol accumulation after herbivory (**Fig 6A**; *F*_1,16_ = 12.319, *P* = 0.003), whereas glandless mutants showed constitutively low and non-inducible gossypol levels (**Fig 6A**; *F*_1,16_ = 0.004, *P* = 0.953). Although HIPV exposure did not enhance gossypol accumulation in the gossypol-free mutant plants, both JA and JA-Ile contents were still significantly increased (**S3 Fig**; JA: *F*_1,12_ = 5.082, *P* = 0.044; JA-Ile: *F*_1,12_ = 5.963 *P* = 0.031), confirming that the JA signaling pathway acts upstream in regulating gossypol metabolic pathway. Correspondingly, HIPV exposure significantly reduced CBW larval weight only in WT plants (**Fig 6B**; *F*_1,44_ = 7.790, *P* = 0.008), with no effect in mutants (**Fig 6B**; *F*_1,44_ = 0.115, *P* = 0.736). Together, these data demonstrate that HIPV-primed resistance in cotton requires gland-dependent gossypol-mediated defense.

## Discussion

Plants have evolved sophisticated mechanisms to counter diverse threats, and HIPVs/OIPVs represent a key mechanism enabling plants to anticipate and counter herbivore attacks [44–47]. Priming neighboring plants through volatile cues is widely viewed as an energy-efficient defensive strategy [48]. Here, we demonstrate that HIPVs emitted by CBW-infested cotton plants prime neighboring plants for enhanced resistance under both laboratory and semi-field conditions (**Fig 1**). By integrating chemical, molecular, and genetic analyses, we further elucidate the phytohormonal pathway and key defensive metabolite underlying this primed resistance.

Our findings demonstrate that exposure to CBW-induced HIPVs alone did not directly activate JA accumulation or the expression of JA signaling genes. Instead, it primed receiver plants for a faster and stronger expression of most JA signaling genes and higher induction of JA levels upon CBW attack (**Fig 2**). This volatile-mediated priming was JA-dependent, as JA biosynthesis inhibitors completely abolished both the enhanced JA burst and the associated resistance (**Fig 3**). Thus, this cotton defensive trait relies on both HIPV exposure and subsequent herbivory, confirming that it is a case of *defensive priming* rather than *defense induction.* Unlike direct induction, priming avoids unnecessary metabolic expenditure while establishing a physiological “memory” that enables rapid defense activation upon attack [20].

The contrasting findings between our study and previous work showing limited HIPV-induced responses in cotton [38] may be explained by differences in cotton varieties or insect species employed. This interpretation is consistent with earlier evidence that both plant genotype and attacker identity can shape the strength of volatile-mediated priming [20]. Notably, cotton may have evolved more specialized priming mechanisms against *H. armigera*, a major long-term pest, compared with *Spodoptera* species such as *S. frugiperda* or *S. exigua*.

Gossypol is a central anti-herbivore compound in cotton, impairing insect digestive function and thereby compromising larval growth [49,50]. We found that HIPV exposure significantly enhanced gossypol accumulation after herbivory (**Fig 4**), and this effect was entirely absent in glandless *gl*_*2*_*gl*_*2*_*gl*_*3*_*gl*_*3*_ mutant (**Fig 5**,**6**). However, the activation of the JA signaling pathway by HIPV exposure remained unaffected in the glandless mutant (**S3 Fig**). These results further confirmed previous studies that JA signaling pathway acts upstream in regulating gossypol metabolic pathway [37]. Although gossypol is likely the principal metabolite driving HIPV-primed resistance, given its well-established importance in defense against CBW [42], we cannot fully exclude the possibility that other compounds, such as hemigossypolon and heliocides, also contribute to the observed resistance effects. Nonetheless, collectively, these results support a model in which HIPV perception sensitizes the JA pathway, enabling rapid gossypol biosynthesis upon herbivore attack. This model is consistent with prior findings that methyl jasmonate treatment boosts gossypol production [51] and that overexpression of *GhMYC2* enhances gossypol accumulation [43].

From an applied perspective, cotton remains highly vulnerable to herbivorous pests, and reliance on synthetic pesticides threatens both environmental and economic sustainability [52]. In this context, volatile-mediated ecological regulation represents a promising avenue for sustainable pest management [8]. A number of volatiles have already been identified as defense elicitors in other crops. For instance, exposure to indole enhances resistance against herbivores in rice, maize, and tea plants [22,23,53], while DMNT exposure improves defenses in tea and sweet potato [24,25]. However, the specific HIPVs that mediate pest resistance in cotton, and their roles in plant communication, remain largely unknown. Identifying these key HIPVs, and their receptors, holds substantial practical importance: it could lead to the development of novel plant elicitors for agricultural use or inform the breeding of insect-resistant cotton varieties, ultimately reducing chemical pesticide dependency.

In conclusion, our study elucidates the mechanism by which HIPVs prime herbivore resistance in cotton, bridging airborne cues to enhanced defense signaling and metabolite biosynthesis. We demonstrate that exposure to HIPVs from CBW-infested plants upregulates JA signaling genes and elevates endogenous JA levels upon subsequent attack, which in turn promotes the biosynthesis of the defensive metabolite gossypol and ultimately strengthens resistance against CBW. Given the dense canopy structure of cotton fields and the prevalence of multiple co-occurring pests, such priming mechanisms may bear widespread ecological significance. Understanding the molecular and ecological foundations of volatile-mediated communication not only advances fundamental plant biology but also provides a foundation for harnessing airborne alarm cues in sustainable pest management strategies.

## Materials and methods

### Plants and insects

Cotton plants (*Gossypium hirsutum* L., cultivar TM-1) were grown in a glasshouse at 26 ± 2 °C, 50 ± 10% relative humidity (RH) and a 14:10 h light:dark photoperiod. Prior to sowing, cotton seeds were soaked in 37 °C warm water for 5 h and then planted in plastic pots (d = 8 cm, h = 8 cm) filled with a 2:2:1 (v/v/v) mixture of substrate, peat, and vermiculite. Seedlings were watered weekly, without fertilization. Plants were used at the 4^th^ leaf stage (excluding cotyledons), corresponding to the emergence of the 5^th^ leaf, approximately 4–6 weeks after planting. A glandless cotton line (genotype *gl*_*2*_*gl*_*2*_*gl*_*3*_*gl*_*3*_) in the genetic background of the cotton cultivar ‘Zhongmian 12’ [54,55] was kindly provided by Prof. Changsong Zou (Henan University, China).

The cotton bollworm (*Helicoverpa armigera*; CBW), a cosmopolitan generalist herbivore and major cotton pest [56], was used for all bioassays. CBW larvae were obtained from Keyun Biological Corporation (Jiyuan, China), reared on an artificial diet consisting of soybean powder and wheat bran, and maintained in plastic containers within a controlled greenhouse set at 27 ± 2 °C, 60 ± 10% RH and a photoperiod of 16:8 h light:dark.

### Experimental exposure treatment

We employed a push-pull system to expose receiver cotton plants to volatiles emitted by either uninfested plants (Control) or plants that had been infested with CBW (HIPVs), as described by Yao et al. (2023) [26]. Herbivore-induced emitter plants were infested with two starved 3^rd^-instar CBW larvae for 24 h, whereas control emitters remained undamaged. Charcoal-purified air was pushed into the emitter vessel (d = 22 cm, h = 45 cm) and directed through a Teflon tube to the receiver vessel at a flow rate of 600 mL/min. After 48 h of continuous exposure, the receiver plants were used for CBW larval performance bioassays, and leaf samples were collected for subsequent analyses, including gene expression, phytohormone levels, and gossypol content.

### Performance of caterpillars on volatiles-exposed plants

*Indoor assays* - Receiver plants were exposed to HIPVs or control volatiles for 48 h and then each plant was infested with four uniformly sized neonate larvae (assay 1) or four 2-day-old larvae (assay 2). Larvae fed for 72 h, after which individual fresh weight was measured (Sartorius, Beijing, China). The average weight per plant represented one biological replicate (*n* = 13 for neonates; *n* = 9 for 2-day-old larvae). All assays were conducted at 26 ± 2 °C, 60 ± 5% RH, and a 16:8 h L:D photoperiod.

*Semi-field assays -* Semi-field trials were conducted outdoors in Kaifeng, China (34.49 °N, 114.18 °E) during July–October. Greenhouse-grown 4-leaf plants were transplanted into insect-exclusion cages (outer cage: 0.6 × 0.6 × 0.7 m, 120-mesh). A smaller inner cage (0.3 × 0.3 × 0.6 m, 80-mesh) contained two receiver plants. Eight emitter plants (uninfested or infested for 24 h with two starved 3^rd^-instar larvae) were arranged around the inner cage. After 24 h of volatile exposure, each receiver plant was infested with eight neonate larvae for 72 h. Mean larval weight per plant was used as one replicate. Three independent batches yielded 20 replicates per treatment.

### RNA isolation, cDNA synthesis and real-time quantitative PCR analysis

Leaf samples were collected from receiver plants after 48 h of exposure to HIPVs from uninfested or CBW-infested cotton plants. Sampling was performed at three time points: immediately after exposure (0 h, defense induction), or after subsequent infestation with two 2^nd^-instar CBW larvae for 3 h or 24 h (defense priming). For each time point and treatment, leaves from three plants were pooled to form one biological replicate, with four replicates collected per condition. Frozen leaf tissue was ground in liquid nitrogen, and total RNA was extracted using the M5 Plant RNeasy Complex Mini Kit (Mei5bio, Beijing, China). RNA concentration was determined on a NanoDrop One spectrophotometer (Thermo Scientific). cDNA was synthesized from 1000 ng of total RNA using the PrimeScript RT Reagent Kit with gDNA Eraser (Takara) according to the manufacturer’s instructions.

Transcript levels of eight jasmonic acid (JA)-related genes were quantified by real-time quantitative PCR (RT-qPCR) on a LightCycler 480 II system (Roche Diagnostics GmbH, Switzerland). Each 10 µL reaction contained 2 µL cDNA, 5 µL of 2 × M5 HiPer SYBR Premix Es Taq (Mei5bio), 0.2 µL each of forward and reverse primers (10 µM), and 2.6 µL nuclease-free water. The PCR protocol consisted of initial denaturation at 95 °C for 10 min, 40 cycles of 95 °C for 15 s and 60 °C for 1 min, followed by melting curve analysis from 65 °C to 95 °C. Gene-specific primers (**S1 Table**) were designed using primer 5 software (Premier Biosoft International, Palo Alto CA, USA). Relative expression was calculated by the 2^-ΔCt^ method, with *Histone3* as the reference gene.

### Plant hormones extraction and analysis

After a 48-hour exposure to volatiles from either uninfested or CBW-infested cotton plants, receiver plants were infested with two 2^nd^-instar CBW larvae for 0, 3, 6, 12, or 24 h prior to leaf tissue collection. The harvested leaves were flash-frozen in liquid nitrogen and ground into a fine powder. Approximately 150 mg of each powdered sample was placed on dry ice and shipped to the analytical platform at the Kunming Institute of Botany, Chinese Academy of Sciences (Kunming, China) for phytohormone analysis. The contents of JA and JA-Ile were quantified by comparing their chromatographic peak areas with those of corresponding internal standards, following the method described by Jin et al. (2023) [57]. Four biological replicates were performed per treatment.

### Determination of phytohormone and gossypol levels, as well as CBW performance in cotton under volatiles exposure and JA synthesis inhibitors treatment

To assess the role of JA signaling in HIPV-mediated priming, cotton plants were treated with the lipoxygenase inhibitor salicylhydroxamic acid (SHAM) and the allene oxide synthase inhibitor diethyldithiocarbamic acid (DIECA), both obtained from Sigma Chemical Company (St. Louis, MO). These inhibitors were applied at concentrations of 200 μM SHAM and 100 μM DIECA, as established in previous study [53]. Receiver plants were sprayed uniformly using a vaporizer at two time points: 3 h before and immediately after volatile exposure, to ensure effective suppression of JA biosynthesis. Control plants received distilled water containing 0.02% (v/v) Tween-20.

After exposure for 48 h to uninfested or CBW-induced volatiles, inhibitor-treated and untreated receiver plants were infested with two 2^nd^-instar CBW larvae for 24 h before leaf tissue collection. JA and JA-Ile levels were then quantified, with six biological replicates per treatment. In parallel, CBW larval performance was evaluated on similarly prepared plants. Following volatile exposure and buffer or inhibitor treatments, each receiver plant was infested with four uniformly sized 2-day-old CBW larvae. After 72 h of feeding, the mean larval weight per plant was recorded. Twelve biological replicates were performed for each treatment condition.

To verify that the JA signaling pathway indeed serves as the causal mediator linking volatile perception to increased gossypol production, we further quantified gossypol accumulation in the receiver plants that were subjected to the same treatment procedures as described above. The plant tissue samples were harvested after 36 h of CBW feeding. Four samples (replicates) were included for each treatment, and gossypol levels were quantified using the method as described below.

### Gossypol extraction and analysis

Following a 48 h exposure to volatiles from either uninfested or CBW-infested cotton plants, receiver plants were infested with two 2^nd^-instar CBW larvae for 0, 24, and 36 h. Leaf tissues were then harvested, immediately frozen in liquid nitrogen, and ground into a fine powder. Approximately 100 mg of powder per sample was extracted with 1 mL of extraction solution containing 80% acetonitrile, 20% water, and 0.5% formic acid. The mixture was sonicated for 10 min, shaken for 10 min, and centrifuged at 12,000 rpm for 10 min. The supernatant was collected, and the pellet was re-extracted with 0.5 mL acetone followed by centrifugation. The combined supernatants were transferred to glass vials for HPLC-MS/MS analysis (LCMS-8040, Shimadzu).

Chromatographic separation was achieved using a Shim-pack XR-ODS III column (2.0 mm × 75 mm, 1.6 μm) maintained at 25 °C. The mobile phase was delivered at a flow rate of 0.3 mL/min, and the injection volume was 10 μL. Gossypol was detected by electrospray ionization in negative mode using multiple reaction monitoring (MRM), with the parent ion detected at *m/z* 517 and the product ion at *m/z* 259.

### Quantification of gossypol and phytohormone levels, as well as CBW performance in glandless cotton mutants following volatiles exposure

To investigate the role of gossypol glands in cotton defense against CBW, we compared the glandless cotton mutant (*gl*_*2*_*gl*_*2*_*gl*_*3*_*gl*_*3*_) with its wild-type (WT) counterpart, Zhongmian12. We first measured gossypol content and assessed CBW performance on both WT and mutant plants. For gossypol quantification, leaf tissues from three plants were pooled as one biological sample, with four replicates per line. Extraction and quantification followed previously described methods. For larval performance assays, each plant was infested with four uniformly sized neonates and allowed to feed for 72 h, after which larval weight was recorded. Ten replicates were included per genotype.

We further examined the effect of HIPV exposure on both WT and *gl*_*2*_*gl*_*2*_*gl*_*3*_*gl*_*3*_ mutant plants. For larval performance and gossypol quantification, after exposure for 48 h to volatiles from either uninfested or CBW-induced WT emitters, each receiver plant was infested with four uniformly sized neonates of CBW for 72 h. Larval performance was evaluated by measuring the mean weight of CBW larvae per plant, with twelve replicates per treatment. Immediately following the bioassay, plant tissues were harvested for gossypol quantification. For this analysis, leaves from three plants were combined into one sample, generating five biological replicates per treatment.

To further elucidate the interaction between the JA signaling pathway and gossypol biosynthesis, we measured JA and JA-Ile levels in WT and gossypol-free mutant plants. Plants were exposed for 48 h to volatiles emitted from either uninfested or CBW-infested WT plants. Subsequently they were infested with two 2^nd^-instar CBW larvae for 24 h before plant tissues were sampled. Four replicates were included for each treatment and JA and JA-Ile contents were determined as described above.

### Statistical analysis

Prior to analysis, all datasets were checked for normality using the Shapiro-Wilk test and for homogeneity of variance using Levene’s test. Specific statistical models were applied as follows:

Data for CBW larval weight and gossypol content between treatments were analyzed using two-sided Student’s *t*-test, except for larval weight in the semi-field assay, which were fitted using linear mixed-effects models with test batch included as a random factor, followed by Bonferroni-corrected pairwise comparisons.

Gene expression, phytohormone, and gossypol levels were evaluated by two-way analysis of variance (ANOVA), with volatile exposure treatment and herbivore infestation duration as independent variables, followed by Bonferroni-adjusted pairwise comparisons.

Data for phytohormone and gossypol levels, as well as CBW weight value between exposed-WT and -mutants or exposed-untreated and inhibitor-treated plants were analyzed using two-way ANOVA, with exposure treatment and cotton line/treatment as independent variables, followed by pairwise comparisons through Bonferroni adjustment. All analyses were performed using SPSS 26.0 (IBM SPSS, Somers, NY, USA).

## Supporting information

S1 Fig Other JA-related genes expression in receiver plants. Expression profiles of two JA signaling-related genes in receiving plants that had been exposed for 48 h to volatiles from uninfested or CBW-infested plants following CBW caterpillar infestation at different time points. Gene abbreviations: *GhOPR2*, *G. hirsutum* 12-oxophytodienoate reductase 2; *GhLOX1*, *G. hirsutum* lipoxygenase 1. Bars represent mean ± SE (*n* = 4; two-way ANOVA followed by pairwise comparisons through Bonferroni adjustment; ***, *P* < 0.001).
(TIF)

S2 Fig Gossypol content in JA inhibitors-treated cotton plants exposed to HIPVs prior to CBW feeding. The receiver plants were pre-exposed for 48 h to volatiles emitted from either uninfested or CBW-infested cotton plants. They were then treated with either buffer (Tween-20) or JA synthesis inhibitors (SHAM and DIECA), followed by CBW infestation for 36 h. Asterisks indicate significant differences between treatments (*n* = 4; two-way ANOVA followed by pairwise comparisons through Bonferroni adjustment; **, *P* < 0.01).
(TIF)

S3 Fig JA and JA-Ile contents in WT plants and gossypol-free mutants under HIPV exposure and CBW feeding. Contents of JA **(A)** and JA-Ile **(B)** in WT or *gl*_*2*_*gl*_*2*_*gl*_*3*_*gl*_*3*_ mutant plants that had been pre-exposed for 48 h to volatiles from either uninfested or CBW-infested WT plants followed by CBW caterpillar infestation for 24 h. Asterisks indicate significant differences between treatments (*n* = 4; two-way ANOVA followed by pairwise comparisons through Bonferroni adjustment; *, *P* < 0.05).
(TIF)

S1 Table Genes and primer pairs used for RT-qPCR in the study. (XLSX)

S1 Data Complete raw numerical dataset underlying all data graphs shown in the manuscript. (XLSX)
