# Supplementary figures and images for: Exposure to herbivore-induced plant volatiles primes JA-dependent gossypol defenses in cotton
Source: PLoS Pathog. 2026 Jun 11;22(6):e1014338. doi: 10.1371/journal.ppat.1014338 (PMC13286275; doi:10.1371/journal.ppat.1014338)

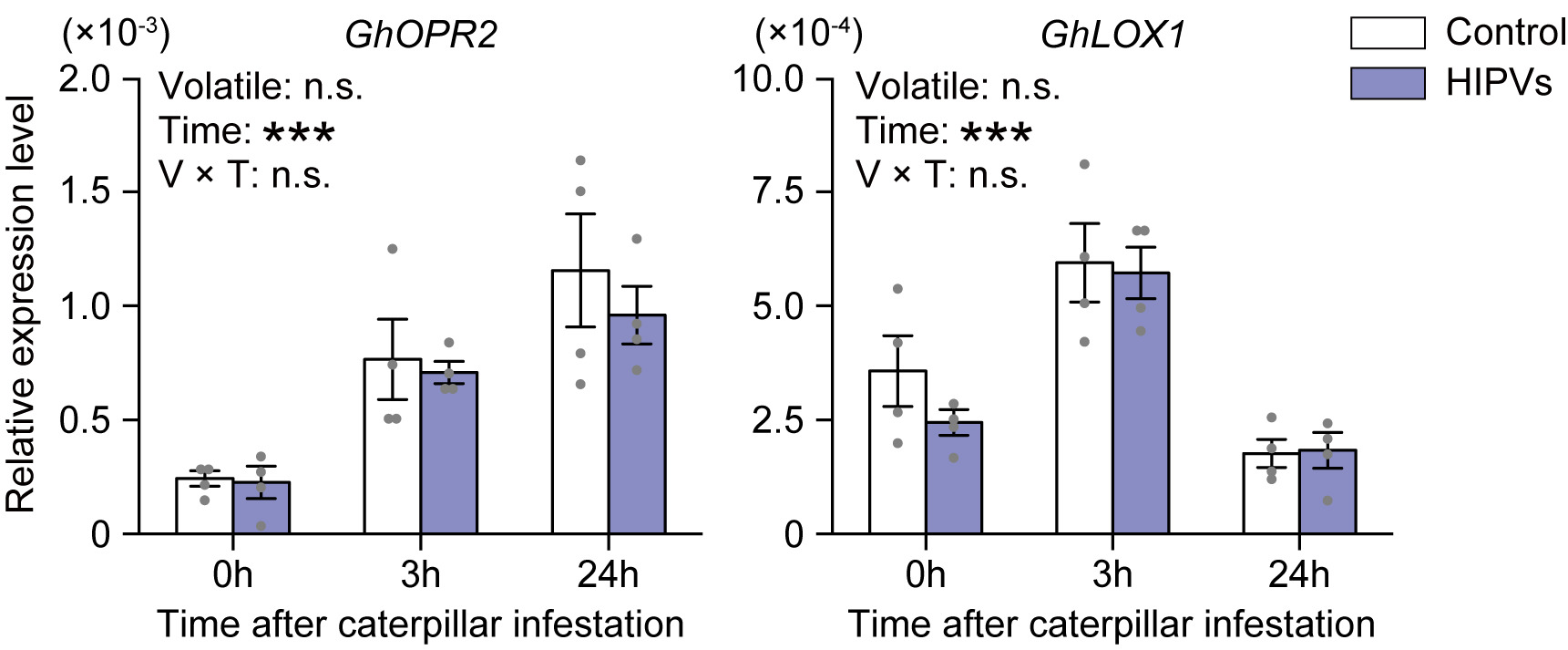

Supplement: S1 Fig — Expression profiles of two JA signaling-related genes in receiving plants that had been exposed for 48 h to volatiles from uninfested or CBW-infested plants following CBW caterpillar infestation at different time points. Gene abbreviations: GhOPR2, G. hirsutum 12-oxophytodienoate reductase 2; GhLOX1, G. hirsutum lipoxygenase 1. Bars represent mean ± SE (n = 4; two-way ANOVA followed by pairwise comparisons through Bonferroni adjustment; ***, P < 0.001). (TIF) [file ppat.1014338.s001.tif]

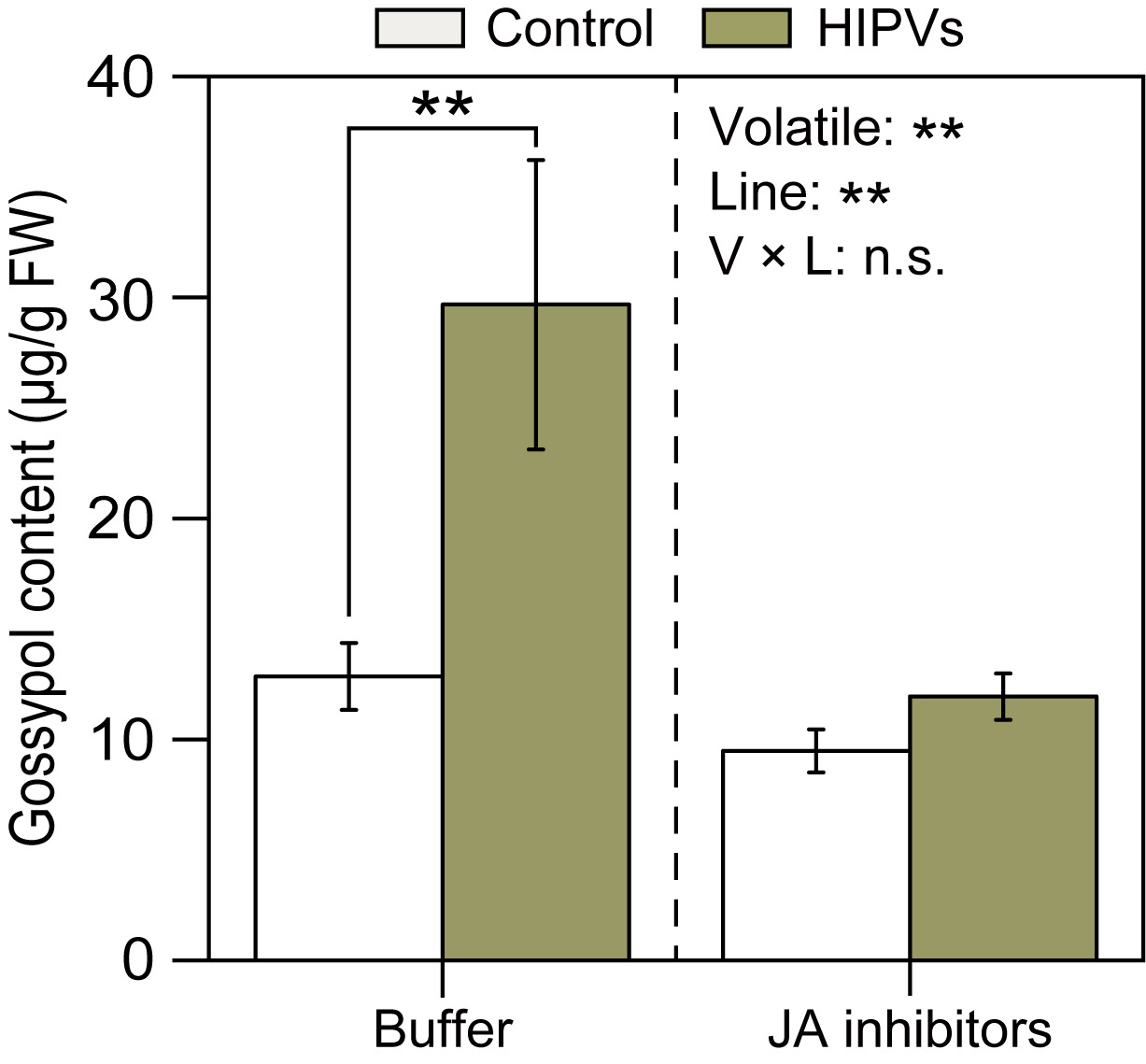

Supplement: S2 Fig — The receiver plants were pre-exposed for 48 h to volatiles emitted from either uninfested or CBW-infested cotton plants. They were then treated with either buffer (Tween-20) or JA synthesis inhibitors (SHAM and DIECA), followed by CBW infestation for 36 h. Asterisks indicate significant differences between treatments (n = 4; two-way ANOVA followed by pairwise comparisons through Bonferroni adjustment; **, P < 0.01). (TIF) [file ppat.1014338.s002.tif]

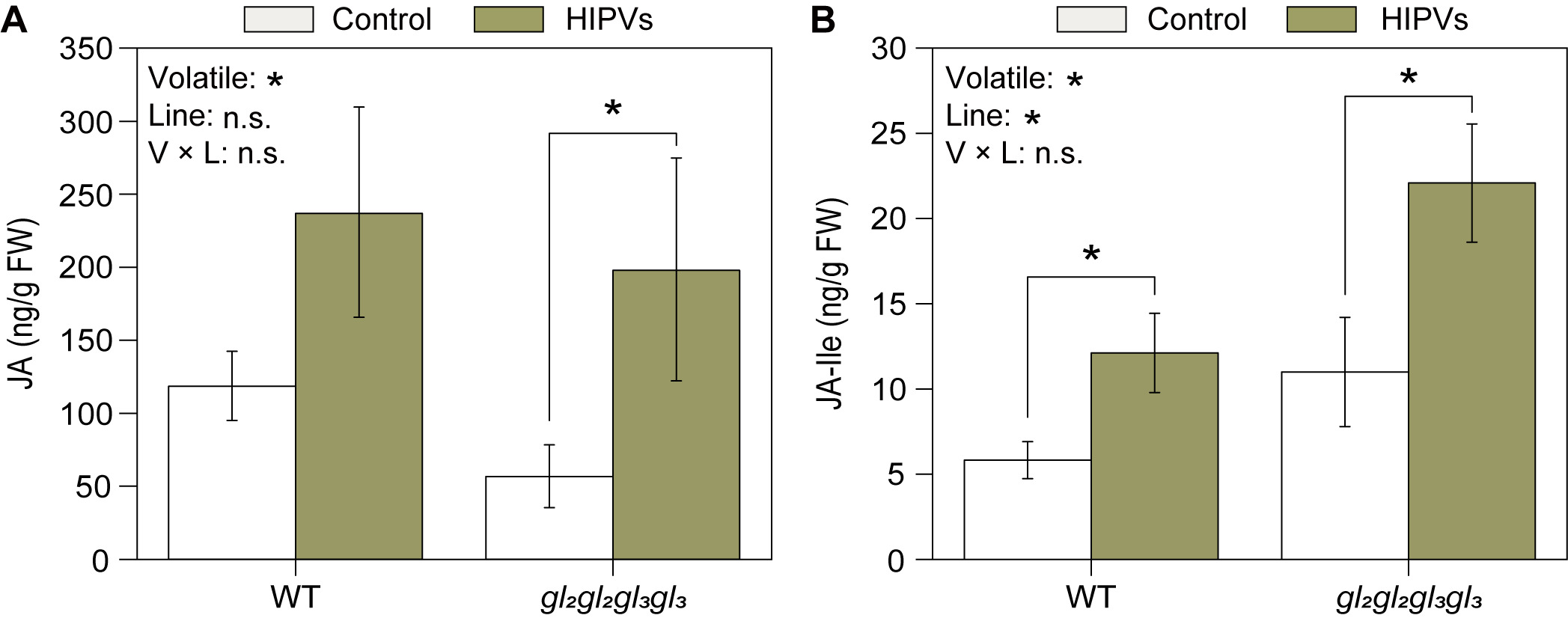

Supplement: S3 Fig — Contents of JA (A) and JA-Ile (B) in WT or gl2gl2gl3gl3 mutant plants that had been pre-exposed for 48 h to volatiles from either uninfested or CBW-infested WT plants followed by CBW caterpillar infestation for 24 h. Asterisks indicate significant differences between treatments (n = 4; two-way ANOVA followed by pairwise comparisons through Bonferroni adjustment; *, P < 0.05). (TIF) [file ppat.1014338.s003.tif]
