# Supplementary material for: Exposure to herbivore-induced plant volatiles primes JA-dependent gossypol defenses in cotton
Source: PLoS Pathog. 2026 Jun 11;22(6):e1014338. doi: 10.1371/journal.ppat.1014338 (PMC13286275; doi:10.1371/journal.ppat.1014338)
Supplement: Attachment — Submitted filename: Response to Reviewers.docx [file ppat.1014338.s006.docx]

Dear reviewers,

We are grateful for the positive and constructive review of our manuscript. We have carefully revised the manuscript in accordance with your suggestions and comments, and we describe in detail below how we have addressed each point you raised (in blue).

Kind regards,

Yunhe Li

Reviewer #1: This manuscript by Zhu et al. demonstrates that cotton bollworm-induced plant volatiles can prime the defense responses of neighboring plants. Specifically, exposure to these volatiles enhances the accumulation of herbivory-induced jasmonic acid and the biosynthesis of the defensive metabolite gossypol.

Reviewer #2: Zhu et al. demonstrated that cotton bollworm-induced volatiles can serve as defense-priming stimuli in neighboring cotton plants, both in laboratory and semi-field conditions. These volatiles were shown to prime JA signaling and promote gossypol accumulation in neighboring plants. Furthermore, the priming effect was abolished when JA signaling or gossypol biosynthesis was inhibited in the receiver plants. Given that volatile-mediated signaling is known to be context- and species-specific, studies conducted in crop systems such as cotton are of particular interest. I have only a few minor suggestions for this otherwise well-written manuscript.

R: Thanks to the reviewer for the positive comments on our MS.

Reviewer #3: Zhu et al. base their work on practical agricultural production needs and employ an experimental system combining laboratory and semi-field conditions. They clearly demonstrate that volatiles induced by cotton bollworm (CBW) infestation enhance the defensive resistance of recipient cotton plants against subsequent herbivore attack, effectively reducing feeding damage. Building on this, the study reveals that CBW-induced volatiles prime anti-herbivore defense in cotton by potentiating the JA signaling pathway and promoting gossypol biosynthesis upon actual herbivore challenge. These findings fill a critical knowledge gap in cotton HIPV communication research by showing how defense signals are transduced into chemical defenses. The regulatory mechanism elucidated here also provides important theoretical support and practical reference for reducing chemical pesticide use in sustainable agriculture and for developing novel biocontrol strategies. However, there are still some drawbacks that should be addressed.

R: Thanks to the reviewer for the positive comments on our MS.

**Major Issues: Key Experiments Required for Acceptance**

Reviewer #1: The study on the gossypol-deficient mutant *gl_2_gl_2_gl_3_gl_3_* clearly demonstrates that gossypol contributes to the volatile-induced priming effect. Given the authors' proposed model that "HIPV perception sensitizes the JA pathway, enabling rapid gossypol biosynthesis upon herbivore attack" (Lines 205-206), I am curious to know whether the enhanced JA induction they observed is also detectable in these gossypol-free lines.

R: To answer the question raised by this reviewer, we conducted an additional experiment in which the gossypol-deficient mutant was exposed to volatiles from uninfested or CBW-infested plants. The results clearly show that pre-exposure of HIPVs can remarkedly enhance the accumulation of JA and JA-Ile upon subsequent herbivory not only in WT cotton but also in gossypol-free cotton line (**S3 Fig.**). This result supports our model that JA pathway can regulate the gossypol metabolic pathway at the upstream level and mediate gossypol biosynthesis, whereas the gossypol metabolic pathway exerts no regulatory effect on the JA pathway. The new data was added into the revised version (see lines 192-195 and S3 Fig.).

Reviewer #2: (No Response)

Reviewer #3: (1) I have concerns regarding the strength of the evidence supporting the conclusion that "CBW-induced volatiles prime anti-herbivore resistance in cotton by potentiating the JA signaling pathway, which in turn enhances gossypol biosynthesis upon actual herbivore attack." While the study successfully demonstrates that volatile exposure activates the JA pathway and promotes gossypol accumulation, a critical gap remains: it does not fully establish that the JA pathway serves as the causal mediator linking volatile perception to increased gossypol production. I recommend either providing additional mechanistic evidence or rephrasing this conclusion to avoid ambiguity.

R: We understand the concern raised by this reviewer.

Since in many previous studies, JA pathway has well confirmed to regulate the gossypol metabolic pathway at the upstream level and mediate gossypol biosynthesis. We therefore did not repeatedly verify this causality. To better address the reviewer’s comment, and further clarify this point, we performed an additional test in which the HIPVs receiver cotton plants were treated with a JA biosynthesis inhibitor before and after HIPVs exposure prior to CBW infestation. As the new data shown in **S2 Fig.**, in the buffer-treated control plants, HIPV exposure significantly increased gossypol accumulation, however in **inhibitor-treated** plants the pre-exposure to HIPVs cannot increase gossypol production. This result well proved that the JA pathway serves as the causal mediator linking volatile perception to increased gossypol production. The new data were incorporated into the revised version (see lines 167-169 and S2 Fig.).

**Minor Issues: Editorial and Data Presentation Modifications**

Reviewer #1: The sample size for the herbivore bioassay (n ≈ 10) is insufficient for a reliable larval growth assay, given the inherent natural variation in such experiments.

R: We fully understand the concern raised by this reviewer.

In our bioassays, although we only set 10 replicates per treatment, each replicate included 4 individual larvae, leading to a total 40 larvae assessed for each treatment. Based on our experience, such a sample size is adequate. Furthermore, we detected the significant differences among treatments, further verifying that the current sample size adopted in this bioassay deliver sufficient statistical power.

Reviewer #2: 1. In the Abstract (line 24), the statement “the molecular basis for this phenomenon remains poorly understood” could be made more specific by explicitly mentioning cotton.

R: We agree with the reviewer that the statement could be more specific. We have therefore revised the sentence in the Abstract to explicitly mention cotton, clarifying that the molecular basis for this phenomenon remains poorly understood, particularly in cotton.

2. The results for the first (line 105) and third (line 133) main experiments are described too briefly. Please add clarifying details to these sections, such as a short description of the experimental design and the names of the JA inhibitors used.

R: Since the experimental design and the names of JA inhibitors have been clearly described in the Materials and Methods section, we originally presented only the results here without redundant description. Nevertheless, we agree with the reviewer that relevant information at this location can help readers better understand the results. Accordingly, we have added the corresponding information following the reviewer’s suggestion (see lines 156-158).

3. In Figure 1, the number of replicates differs between the Figure legend and the Methods section. For instance, the lab assays used “four uniformly sized CBW larvae,” yet the replicates were 13 or 8-9. A similar discrepancy appears in the semi-field assay data.

R: Thanks to reviewer for careful review on our MS.

However, we believe there is a misunderstanding on this point. The phrase “four uniformly sized CBW larvae” in the Methods section refers to the larval number placed on each individual plant (i.e., each replicate), whereas the values of n=13 or 8-9 in the figure legend indicate the number of biological replicates (individual plants). We have revised the corresponding sentences in the manuscript to clarify this distinction explicitly.

4. In Figure 2B, CBW-induced JA-Ile levels showed no significant difference between the control and HIPV-treated groups at 24 h treatment. However, Figure 3B demonstrates a significant increase in JA-Ile in the HIPV-treated group. Please discuss this discrepancy.

R: Thanks to this review for careful review on our MS.

Based our experience with hormone measurements and data from previous studies (Ye et al., 2019, Plant Cell; Yao et al., 2023, New Phytol), the discrepancy in JA-lle levels between our two bioassays is in fact reasonable and expected. JA is a **transient, rapidly regulated signaling molecule**, and its accumulation in plant is highly time‑dependent and susceptible to subtle variations in plant growth status, environmental conditions and physiological synchrony. Although the plant tissues were sampled at the same time point after being exposed to HIPVs, it is not possible to ensure that the plant physiology were identical across the two separate independent experiments.

In particular, JA-Ile is the bioactive form of JA, which is converted from JA via catalysis by *JAR1*. The ratio of JA and JA-Ile changes dynamically and rapidly when plant responses to biotic or abiotic stresses. Thus, variations in content of both JA and JA-Ile, or single JA or JA-lle can collectively reflect the functional status of the JA signaling pathway. Although the change in JA-Ile did not reach statistical significance, a clear increasing trend was still observed in Figure 2B. More importantly, we did detect a significant increase in JA content in HIPV-exposed cotton plants. This result can well support our main conclusion that JA signaling pathway play the main role for regulating CBW resistance in receiver cotton plants even if there is no significance was not detected for JA-Ile content.

5. Although it is well established that gossypol biosynthesis is regulated by JA signaling, to further support the conclusion that primed JA signaling correlates with gossypol biosynthesis in neighboring plants, I recommend either: (1) quantifying gossypol accumulation in receiver plants treated with buffer or JA inhibitors, or (2) examining the expression of gossypol biosynthesis-related genes in HIPV-exposed plants versus control plants treated with CBW, as illustrated in Fig. 2.

R: As we mentioned above and the comment by this reviewer, JA pathway has well established to regulate the gossypol metabolic pathway at the upstream level and mediate gossypol biosynthesis (Han et al., 2022). We therefore did not repeatedly verify this causality. Even so, according to the suggestion raised by this reviewer, we performed an additional test in which the HIPVs receiver cotton plants were treated with a JA biosynthesis inhibitor before and after HIPVs exposure prior to CBW infestation. The result clearly showed that the JA pathway serves as the causal mediator linking volatile perception to increased gossypol production (**S2 Fig.**). The new data were incorporated into the revised version (lines 167-169).

Reviewer #3: I recommend improving the logical coherence of the Introduction section. For example, in Lines 65-80, the text abruptly transitions from introducing constitutive defense compounds (pigment glands, gossypol) that can be induced by pests to discussing HIPV-induced defense responses. It is suggested to clarify the relationship between HIPV-mediated jasmonic acid (JA) signaling pathway and downstream defense compounds (such as gossypol) to enhance the logical flow of the Introduction.

R: We fully agree to this comment.

The sentences were significantly modified to better show the relationship between HIPV-mediated JA signaling and the downstream defense compounds (see lines 90-94) in the revised version.

Given that gene expression, phytohormone, and gossypol levels were evaluated by two-way ANOVA, the figures should therefore present the results of this analysis. Please include the P values for treatment effect, time effect, and their interaction in each figure panel or legend.

R: We thank the reviewer for this constructive suggestion. We have revised all relevant figures to include the two-way ANOVA results as suggested. Specifically, the results of the analysis of the treatment effect, time effect, and their interaction are now presented in each figure panel for all assays, including gene expression, phytohormone, and gossypol level measurements.

Line 72：Please add the definite article "the" before "lepidopteran larva" .

R: Done as suggested.

Line 87: correct the typo "plans" to "plants".

R: Thank you for pointing out this typo. It was corrected in the revised version.

Line 109 and line 158: Please confirm that the degrees of freedom (df) are integers, and unify the number of decimal places throughout the manuscript.

R: We greatly appreciate the reviewer for pointing out the mistakes regarding the degrees of freedom. We corrected the errors in the revised version.

In Figure 1: Only the final body weight of cotton bollworm (CBW) was presented in Figures 1B and 1D. Even with uniform initial selection, the milligram-scale body weight of the larvae means that minor initial differences could have affected the results. It is recommended that authors record the initial larval weight and use weight gain as the evaluation index to improve the reliability of the results.

R: We fully agree with the reviewer that measuring initial larval weight and using weight gain as an evaluation index would improve the reliability of the results. However, this approach is more feasible for relatively large insect larvae. In the present study, we used newly hatched neonate cotton bollworm larvae, which are extremely tiny. Even a 0.00001 g analytical balance cannot generate highly accurate mass readings for such small individuals.

Moreover, manipulating these tender neonates for weighing inevitably causes mechanical injury, which would introduce experimental bias and compromise the validity of the bioassay. Based on our experience, selecting uniformly sized larvae without measuring initial body weight is a more reliable approach to reflect the actual treatment effects on performance of such small size larvae.

In Figure 2: The data for JA-Ile at 12 h show high variability. I recommend verifying these results. Furthermore, JA pathway-related gene expression was only measured at 3 h and 24 h, whereas significant differences in JA content were observed at 12 h and 24 h. To address this temporal gap, including gene expression data at or before the 12 h time point is suggested.

R: We understand the concern raised by this reviewer.

Phytohormones as transient signaling molecules, and their levels fluctuate markedly in plant tissues after herbivore infestation. Meanwhile, plant gene expression responds very rapidly upon insect attack. To precisely characterize the temporal dynamics of hormone accumulation, dense time‑point sampling is required, especially during the early stage of infestation. We fully agree with the reviewer’s suggestion that adding sampling time points at or before 12 h would help better interpret the molecular response.

Nevertheless, the biological function of the JA signaling pathway is ultimately executed through hormone metabolites. Therefore, our study places greater emphasis on profiling hormone abundance. For this purpose, we set up five sampling time points within 0–24 h to monitor JA and JA-Ile dynamic changes in concentration. In contrast, the detection of JA pathway-related gene expression was only intended to further confirm the overall response pattern, and the gene expression dynamics is not our focus, therefore we selected only three representative time points: 0 h, 3 h and 24 h.

The current time‑series data of both hormone levels and gene expression fully match the original objectives of our experiment and sufficiently support our core conclusions. Accordingly, we respectfully consider it unnecessary to add extra gene expression sampling points at 12 h. We sincerely appreciate the reviewer’s valuable comment and kindly ask for your understanding.

Line 237: Please clarify the exact temperature of warm water in the Methods.

R: Done as suggested.

Lines 295-296: The relative gene expression was calculated using the 2^-△Ct^ method. Please use the 2^-△△Ct^ method to ensure valid and reliable results.

R: Thanks for careful review on our MS.

We agree that the 2^−ΔΔCt^ method is widely used to calculate relative gene expression when simply comparing each treatment group with a control group. However, in the present study, we adopted the 2^−ΔCt^ method, which directly presents gene expression levels normalized to the reference gene. This approach is well suited to our experimental design, because we applied two‑way ANOVA to evaluate the effects of two independent factors (HIPV exposure and herbivory) as well as their interaction, rather than merely comparing each treatment with a single calibrator sample.

Furthermore, the 2^−ΔCt^ and 2^−ΔΔCt^ methods produce comparable results for treatment-versus-control comparisons, differing only in data scaling and presentation format. The 2^−ΔCt^ approach has also been extensively adopted in previous qPCR studies (Zhang et al., 2019, PNAS; Yao et al., 2025, Current Biology), validating its reliability for such statistical analyses.

**References:**

1. Ye M, Glauser G, Lou YG, Erb M, Hu LF. Molecular dissection of early defense signaling underlying volatile-mediated defense regulation and herbivore resistance in rice. Plant Cell 2019;31(3):687–698. <https://doi.org/10.1105/tpc.18.00569>. PMID: 30760558
2. Yao CC, Du LX, Liu QS, Hu XY, Ye WF, Turlings TCJ, et al. Stemborer-induced rice plant volatiles boost direct and indirect resistance in neighboring plants. New Phytol. 2023;237(6):2375–2387. <https://doi.org/10.1111/nph.18548>. PMID: 36259093
3. Han XP, Xing YD, Zhu YQ, Luo L, Liu LL, Zhai YH, et al. GhMYC2 activates cytochrome P450 gene CYP71BE79 to regulate gossypol biosynthesis in cotton. Planta 2022;256(3):63. <https://doi.org/10.1007/s00425-022-03974-4>. PMID: 35995890
4. Zhang PJ, Wei JN, Zhao C, Zhang YF, Li CY, Liu SS, et al. Airborne host-plant manipulation by whiteflies via an inducible blend of plant volatiles. Proc. Natl. Acad. Sci. USA 2019;116(15):7387–7396. <https://doi.org/10.1073/pnas.1818599116>. PMID: 30910967
5. Yao CC, Zhao X, Hu XY, Rasmann S, Shen J, Turlings TCJ, et al. Planthopper-induced volatiles suppress rice plant defense by targeting *Os4CL5*-dependent phenolamide biosynthesis. Curr Biol. 2025;35(14):3440-3450.e4. <https://doi.org/10.1016/j.cub.2025.06.033>. PMID: 40639375
