# Supplementary material for: Exposure to herbivore-induced plant volatiles primes JA-dependent gossypol defenses in cotton
Source: PLoS Pathog. 2026 Jun 11;22(6):e1014338. doi: 10.1371/journal.ppat.1014338 (PMC13286275; doi:10.1371/journal.ppat.1014338)
Supplement: Peer review history

# Author comment: Author response to Decision Letter 0

**DOI:** 10.1371/journal.ppat.1014338.r001

**Transfer Alert**

This paper was transferred from another journal. As a result, its full editorial history (including decision letters, peer reviews and author responses) may not be present.

18 Jan 2026

# Peer review: Decision Letter 0

**DOI:** 10.1371/journal.ppat.1014338.r002

**Authors:** Melissa Bredow

16 Mar 2026

PPATHOGENS-D-26-00129

Exposure to herbivore-induced plant volatiles primes JA-dependent gossypol defenses in cotton

PLOS Pathogens

Dear Dr. Li,

Thank you for submitting your manuscript to PLOS Pathogens. After careful consideration, we feel that it has merit but does not fully meet PLOS Pathogens's publication criteria as it currently stands. Therefore, we invite you to submit a revised version of the manuscript that addresses the points raised during the review process.

Please submit your revised manuscript by May 15th, 2026. If you will need more time than this to complete your revisions, please reply to this message or contact the journal office at plospathogens@plos.org. When you're ready to submit your revision, log on to https://www.editorialmanager.com/ppathogens/ and select the 'Submissions Needing Revision' folder to locate your manuscript file.

Please include the following items when submitting your revised manuscript:

* A letter that responds to each point raised by the editor and reviewer(s). You should upload this letter as a separate file labeled 'Response to Reviewers'. This file does not need to include responses to any formatting updates and technical items listed in the 'Journal Requirements' section below.

* A marked-up copy of your manuscript that highlights changes made to the original version. You should upload this as a separate file labeled 'Revised Manuscript with Track Changes'.

* An unmarked version of your revised paper without tracked changes. You should upload this as a separate file labeled 'Manuscript'.

If you would like to make changes to your financial disclosure, competing interests statement, or data availability statement, please make these updates within the submission form at the time of resubmission. Guidelines for resubmitting your figure files are available below the reviewer comments at the end of this letter.

We look forward to receiving your revised manuscript.

Kind regards,

Melissa Bredow

Academic Editor

PLOS Pathogens

Shou-Wei Ding

Section Editor

PLOS Pathogens

Sumita Bhaduri-McIntosh

Editor-in-Chief

PLOS Pathogens

orcid.org/0000-0003-2946-9497

Michael Malim

Editor-in-Chief

PLOS Pathogens

orcid.org/0000-0002-7699-2064

**Additional Editor Comments :**

Dear Dr. Yunhe Li,

The reviewers uniformly agree that the work presented fills an important knowledge gap in the field and have recommended publication, but also suggest some minor revisions to the manuscript. Therefore, I invite you to respond to the reviewers' comments and revise your manuscript accordingly.

At this time I would also like to remind you of PLOS Pathogens data availability critera and ask that you review these criteria (https://journals.plos.org/plospathogens/s/data-availability) and ensure that you provide all raw data in a repository, or as a supplemental file, if you have not already done so.

**Journal Requirements:**

1) We ask that a manuscript source file is provided at Revision. Please upload your manuscript file as a .doc, .docx, .rtf or .tex. If you are providing a .tex file, please upload it under the item type u2018LaTeX Source Fileu2019 and leave your .pdf version as the item type u2018Manuscriptu2019.

2) Please provide an Author Summary. This should appear in your manuscript between the Abstract (if applicable) and the Introduction, and should be 150-200 words long. The aim should be to make your findings accessible to a wide audience that includes both scientists and non-scientists. Sample summaries can be found on our website under Submission Guidelines:

https://journals.plos.org/plospathogens/s/submission-guidelines#loc-parts-of-a-submission

3) We do not publish any copyright or trademark symbols that usually accompany proprietary names, eg ©,  ®, or TM  (e.g. next to drug or reagent names). Therefore please remove all instances of trademark/copyright symbols throughout the text, including:

- TM on page: 11.

4) Please upload all main figures as separate Figure files in .tif or .eps format. For more information about how to convert and format your figure files please see our guidelines:

https://journals.plos.org/plospathogens/s/figures

5) We have noticed that you referred to SI Appendix in your manuscript. However, there is no corresponding file uploaded to the submission. Please upload it as a separate file with the item type 'Supporting Information'.

6) We notice that your supplementary Figure, and Table are included in the manuscript file. Please remove them and upload them with the file type 'Supporting Information'. Please ensure that each Supporting Information file has a legend listed in the manuscript after the references list.

7) Some material included in your submission may be copyrighted. According to PLOSu2019s copyright policy, authors who use figures or other material (e.g., graphics, clipart, maps) from another author or copyright holder must demonstrate or obtain permission to publish this material under the Creative Commons Attribution 4.0 International (CC BY 4.0) License used by PLOS journals. Please closely review the details of PLOSu2019s copyright requirements here: PLOS Licenses and Copyright. If you need to request permissions from a copyright holder, you may use PLOS's Copyright Content Permission form.

Please respond directly to this email and provide any known details concerning your material's license terms and permissions required for reuse, even if you have not yet obtained copyright permissions or are unsure of your material's copyright compatibility. Once you have responded and addressed all other outstanding technical requirements, you may resubmit your manuscript within Editorial Manager.

Potential Copyright Issues:

i) Please confirm (a) that you are the photographer of 5A, or (b) provide written permission from the photographer to publish the photo(s) under our CC BY 4.0 license.

ii) Figure 1A. Please confirm whether you drew the images / clip-art within the figure panels by hand. If you did not draw the images, please provide (a) a link to the source of the images or icons and their license / terms of use; or (b) written permission from the copyright holder to publish the images or icons under our CC BY 4.0 license. Alternatively, you may replace the images with open source alternatives. See these open source resources you may use to replace images / clip-art:

- https://commons.wikimedia.org

- https://openclipart.org/.

8) We note that your Data Availability Statement is currently as follows: "All relevant data supporting the findings of this study are included in the article and its Supplemental Information.". Please confirm at this time whether or not your submission contains all raw data required to replicate the results of your study. Authors must share the “minimal data set” for their submission. PLOS defines the minimal data set to consist of the data required to replicate all study findings reported in the article, as well as related metadata and methods (https://journals.plos.org/plosone/s/data-availability#loc-minimal-data-set-definition).

For example, authors should submit the following data:

1) The values behind the means, standard deviations and other measures reported;

2) The values used to build graphs;

3) The points extracted from images for analysis..

Authors do not need to submit their entire data set if only a portion of the data was used in the reported study.

If your submission does not contain these data, please either upload them as Supporting Information files or deposit them to a stable, public repository and provide us with the relevant URLs, DOIs, or accession numbers. For a list of recommended repositories, please see https://journals.plos.org/plosone/s/recommended-repositories.

If there are ethical or legal restrictions on sharing a de-identified data set, please explain them in detail (e.g., data contain potentially sensitive information, data are owned by a third-party organization, etc.) and who has imposed them (e.g., an ethics committee). Please also provide contact information for a data access committee, ethics committee, or other institutional body to which data requests may be sent. If data are owned by a third party, please indicate how others may request data access.

9) Please amend your detailed Financial Disclosure statement. This is published with the article. It must therefore be completed in full sentences and contain the exact wording you wish to be published.

1) State the initials, alongside each funding source, of each author to receive each grant. For example: "This work was supported by the National Institutes of Health (####### to AM; ###### to CJ) and the National Science Foundation (###### to AM)."

2) State what role the funders took in the study. If the funders had no role in your study, please state: "The funders had no role in study design, data collection and analysis, decision to publish, or preparation of the manuscript.".

Note: If the reviewer comments include a recommendation to cite specific previously published works, please review and evaluate these publications to determine whether they are relevant and should be cited. There is no requirement to cite these works unless the editor has indicated otherwise.

**Reviewers' Comments:**

Reviewer's Responses to Questions

**Part I - Summary**

Please use this section to discuss strengths/weaknesses of study, novelty/significance, general execution and scholarship.

Reviewer #1: This manuscript by Zhu et al. demonstrates that cotton bollworm-induced plant volatiles can prime the defense responses of neighboring plants. Specifically, exposure to these volatiles enhances the accumulation of herbivory-induced jasmonic acid and the biosynthesis of the defensive metabolite gossypol.

Reviewer #2: Zhu et al. demonstrated that cotton bollworm-induced volatiles can serve as defense-priming stimuli in neighboring cotton plants, both in laboratory and semi-field conditions. These volatiles were shown to prime JA signaling and promote gossypol accumulation in neighboring plants. Furthermore, the priming effect was abolished when JA signaling or gossypol biosynthesis was inhibited in the receiver plants. Given that volatile-mediated signaling is known to be context- and species-specific, studies conducted in crop systems such as cotton are of particular interest. I have only a few minor suggestions for this otherwise well-written manuscript.

Reviewer #3: Zhu et al. base their work on practical agricultural production needs and employ an experimental system combining laboratory and semi-field conditions. They clearly demonstrate that volatiles induced by cotton bollworm (CBW) infestation enhance the defensive resistance of recipient cotton plants against subsequent herbivore attack, effectively reducing feeding damage. Building on this, the study reveals that CBW-induced volatiles prime anti-herbivore defense in cotton by potentiating the JA signaling pathway and promoting gossypol biosynthesis upon actual herbivore challenge. These findings fill a critical knowledge gap in cotton HIPV communication research by showing how defense signals are transduced into chemical defenses. The regulatory mechanism elucidated here also provides important theoretical support and practical reference for reducing chemical pesticide use in sustainable agriculture and for developing novel biocontrol strategies. However, there are still some drawbacks that should be addressed.

**********

**Part II – Major Issues: Key Experiments Required for Acceptance**

Please use this section to detail the key new experiments or modifications of existing experiments that should be absolutely required to validate study conclusions.

Generally, there should be no more than 3 such required experiments or major modifications for a "Major Revision" recommendation. If more than 3 experiments are necessary to validate the study conclusions, then you are encouraged to recommend "Reject".

Reviewer #1: The study on the gossypol-deficient mutant gl2gl2gl3gl3 clearly demonstrates that gossypol contributes to the volatile-induced priming effect. Given the authors' proposed model that "HIPV perception sensitizes the JA pathway, enabling rapid gossypol biosynthesis upon herbivore attack" (Lines 205-206)， I am curious to know whether the enhanced JA induction they observed is also detectable in these gossypol-free lines.

Reviewer #2: (No Response)

Reviewer #3: (1) I have concerns regarding the strength of the evidence supporting the conclusion that "CBW-induced volatiles prime anti-herbivore resistance in cotton by potentiating the JA signaling pathway, which in turn enhances gossypol biosynthesis upon actual herbivore attack." While the study successfully demonstrates that volatile exposure activates the JA pathway and promotes gossypol accumulation, a critical gap remains: it does not fully establish that the JA pathway serves as the causal mediator linking volatile perception to increased gossypol production. I recommend either providing additional mechanistic evidence or rephrasing this conclusion to avoid ambiguity.

**********

**Part III – Minor Issues: Editorial and Data Presentation Modifications**

Please use this section for editorial suggestions as well as relatively minor modifications of existing data that would enhance clarity.

Reviewer #1: The sample size for the herbivore bioassay (n ≈ 10) is insufficient for a reliable larval growth assay, given the inherent natural variation in such experiments.

Reviewer #2: 1. In the Abstract (line 24), the statement “the molecular basis for this phenomenon remains poorly understood” could be made more specific by explicitly mentioning cotton.

2. The results for the first (line 105) and third (line 133) main experiments are described too briefly. Please add clarifying details to these sections, such as a short description of the experimental design and the names of the JA inhibitors used.

3. In Figure 1, the number of replicates differs between the Figure legend and the Methods section. For instance, the lab assays used “four uniformly sized CBW larvae,” yet the replicates were 13 or 8-9. A similar discrepancy appears in the semi-field assay data.

4. In Figure 2B, CBW-induced JA-Ile levels showed no significant difference between the control and HIPV-treated groups at 24 h treatment. However, Figure 3B demonstrates a significant increase in JA-Ile in the HIPV-treated group. Please discuss this discrepancy.

5. Although it is well established that gossypol biosynthesis is regulated by JA signaling, to further support the conclusion that primed JA signaling correlates with gossypol biosynthesis in neighboring plants, I recommend either: (1) quantifying gossypol accumulation in receiver plants treated with buffer or JA inhibitors, or (2) examining the expression of gossypol biosynthesis-related genes in HIPV-exposed plants versus control plants treated with CBW, as illustrated in Fig. 2.

Reviewer #3: I recommend improving the logical coherence of the Introduction section. For example, in Lines 65-80, the text abruptly transitions from introducing constitutive defense compounds (pigment glands, gossypol) that can be induced by pests to discussing HIPV-induced defense responses. It is suggested to clarify the relationship between HIPV-mediated jasmonic acid (JA) signaling pathway and downstream defense compounds (such as gossypol) to enhance the logical flow of the Introduction.

Given that gene expression, phytohormone, and gossypol levels were evaluated by two-way ANOVA, the figures should therefore present the results of this analysis. Please include the P values for treatment effect, time effect, and their interaction in each figure panel or legend.

Line 72：Please add the definite article "the" before "lepidopteran larva" .

Line 87: correct the typo "plans" to "plants".

Line 109 and line 158 ：Please confirm that the degrees of freedom (df) are integers, and unify the number of decimal places throughout the manuscript.

In Figure 1: Only the final body weight of cotton bollworm (CWB) was presented in Figures 1B and 1D. Even with uniform initial selection, the milligram-scale body weight of the larvae means that minor initial differences could have affected the results. It is recommended that authors record the initial larval weight and use weight gain as the evaluation index to improve the reliability of the results.

In Figure 2: The data for JA-Ile at 12 h show high variability. I recommend verifying these results. Furthermore, JA pathway-related gene expression was only measured at 3 h and 24 h, whereas significant differences in JA content were observed at 12 h and 24 h. To address this temporal gap, including gene expression data at or before the 12 h time point is suggested.

Line 237: Please clarify the exact temperature of warm water in the Methods.

Lines 295-296: The relative gene expression was calculated using the 2-△Ct method. Please use the 2-△△Ct method to ensure valid and reliable results.

**********

PLOS authors have the option to publish the peer review history of their article (what does this mean?). If published, this will include your full peer review and any attached files.

If you choose “no”, your identity will remain anonymous but your review may still be made public.

**Do you want your identity to be public for this peer review?**  For information about this choice, including consent withdrawal, please see our Privacy Policy.

Reviewer #1: No

Reviewer #2: No

Reviewer #3: No

[NOTE: If reviewer comments were submitted as an attachment file, they will be attached to this email and accessible via the submission site. Please log into your account, locate the manuscript record, and check for the action link "View Attachments". If this link does not appear, there are no attachment files.]

**Figure resubmission:**

-->While revising your submission, we strongly recommend that you use PLOS’s NAAS tool (https://ngplosjournals.pagemajik.ai/artanalysis) to test your figure files. NAAS can convert your figure files to the TIFF file type and meet basic requirements (such as print size, resolution), or provide you with a report on issues that do not meet our requirements and that NAAS cannot fix.

After uploading your figures to PLOS’s NAAS tool - https://ngplosjournals.pagemajik.ai/artanalysis, NAAS will process the files provided and display the results in the "Uploaded Files" section of the page as the processing is complete. If the uploaded figures meet our requirements (or NAAS is able to fix the files to meet our requirements), the figure will be marked as "fixed" above. If NAAS is unable to fix the files, a red "failed" label will appear above. When NAAS has confirmed that the figure files meet our requirements, please download the file via the download option, and include these NAAS processed figure files when submitting your revised manuscript.

**Reproducibility:**

To enhance the reproducibility of your results, we recommend that authors of applicable studies deposit laboratory protocols in protocols.io, where a protocol can be assigned its own identifier (DOI) such that it can be cited independently in the future. Additionally, PLOS ONE offers an option to publish peer-reviewed clinical study protocols. Read more information on sharing protocols at https://plos.org/protocols?utm_medium=editorial-email&utm_source=authorletters&utm_campaign=protocols

# Author comment: Author response to Decision Letter 1

**DOI:** 10.1371/journal.ppat.1014338.r003

12 May 2026

Attachment Submitted filename: Response to Reviewers.docx

# Peer review: Decision Letter 1

**DOI:** 10.1371/journal.ppat.1014338.r004

**Authors:** Melissa Bredow

1 Jun 2026

Dear Dr. Li,

We are pleased to inform you that your manuscript 'Exposure to herbivore-induced plant volatiles primes JA-dependent gossypol defenses in cotton' has been provisionally accepted for publication in PLOS Pathogens.

Before your manuscript can be formally accepted you will need to complete some formatting changes, which you will receive in a follow up email. A member of our team will be in touch with a set of requests.

Please note that your manuscript will not be scheduled for publication until you have made the required changes, so a swift response is appreciated.

IMPORTANT: The editorial review process is now complete. PLOS will only permit corrections to spelling, formatting or significant scientific errors from this point onwards. Requests for major changes, or any which affect the scientific understanding of your work, will cause delays to the publication date of your manuscript.

Should you, your institution's press office or the journal office choose to press release your paper, you will automatically be opted out of early publication. We ask that you notify us now if you or your institution is planning to press release the article. All press must be co-ordinated with PLOS.

Thank you again for supporting Open Access publishing; we are looking forward to publishing your work in PLOS Pathogens.

Best regards,

Melissa Bredow

Academic Editor

PLOS Pathogens

Shou-Wei Ding

Section Editor

PLOS Pathogens

Sumita Bhaduri-McIntosh

Editor-in-Chief

PLOS Pathogens

orcid.org/0000-0003-2946-9497

Michael Malim

Editor-in-Chief

PLOS Pathogens

orcid.org/0000-0002-7699-2064

***********************************************************

Reviewer Comments (if any, and for reference):

Reviewer's Responses to Questions

**Part I - Summary**

Please use this section to discuss strengths/weaknesses of study, novelty/significance, general execution and scholarship.

Reviewer #1: Zhu et al. demonstrated that cotton bollworm-induced volatiles can serve as systemic signal trigger neighboring plants' priming effects. This priming effect is depending on JA signaling and gossypol accumulation in neighboring plants. consistently, the priming effect was abolished when JA signaling or gossypol biosynthesis was inhibited in the receiver plants.

Reviewer #2: The authors have satisfactorily addressed all my concerns through additional experiments and revisions. I therefore recommend accepting this manuscript for publication in PLOS Pathogens.

Reviewer #3: The authors have addressed my concerns and the manuscript is acceptable now.

**********

**Part II – Major Issues: Key Experiments Required for Acceptance**

Please use this section to detail the key new experiments or modifications of existing experiments that should be absolutely required to validate study conclusions.

Generally, there should be no more than 3 such required experiments or major modifications for a "Major Revision" recommendation. If more than 3 experiments are necessary to validate the study conclusions, then you are encouraged to recommend "Reject".

Reviewer #1: Authors addressed all my comments, and I don't have further concern.

Reviewer #2: (No Response)

Reviewer #3: NA

**********

**Part III – Minor Issues: Editorial and Data Presentation Modifications**

Please use this section for editorial suggestions as well as relatively minor modifications of existing data that would enhance clarity.

Reviewer #1: (No Response)

Reviewer #2: (No Response)

Reviewer #3: NA

**********

PLOS authors have the option to publish the peer review history of their article (what does this mean?). If published, this will include your full peer review and any attached files.

If you choose “no”, your identity will remain anonymous but your review may still be made public.

**Do you want your identity to be public for this peer review?**  For information about this choice, including consent withdrawal, please see our Privacy Policy.

Reviewer #1: No

Reviewer #2: No

Reviewer #3: No

# Editor report: Acceptance letter

**DOI:** 10.1371/journal.ppat.1014338.r005

**Authors:** Melissa Bredow

Dear Professor Li,

We are delighted to inform you that your manuscript, "Exposure to herbivore-induced plant volatiles primes JA-dependent gossypol defenses in cotton," has been formally accepted for publication in PLOS Pathogens.

We have now passed your article onto the PLOS Production Department who will complete the rest of the pre-publication process. All authors will receive a confirmation email upon publication.

The corresponding author will soon be receiving a typeset proof for review, to ensure errors have not been introduced during production. Please review the PDF proof of your manuscript carefully, as this is the last chance to correct any scientific or type-setting errors. Please note that major changes, or those which affect the scientific understanding of the work, will likely cause delays to the publication date of your manuscript. Note: Proofs for Front Matter articles (Pearls, Reviews, Opinions, etc...) are generated on a different schedule and may not be made available as quickly.

Soon after your final files are uploaded, the early version of your manuscript, if you opted to have an early version of your article, will be published online. The date of the early version will be your article's publication date. The final article will be published to the same URL, and all versions of the paper will be accessible to readers.

For Research Articles, you will receive an invoice from PLOS for your publication fee after your manuscript has reached the completed accept phase. If you receive an email requesting payment before acceptance or for any other service, this may be a phishing scheme. Learn how to identify phishing emails and protect your accounts at https://explore.plos.org/phishing.

Thank you again for supporting open-access publishing; we are looking forward to publishing your work in PLOS Pathogens.

Best regards,

Sumita Bhaduri-McIntosh

Editor-in-Chief

PLOS Pathogens

orcid.org/0000-0003-2946-9497

Michael Malim

Editor-in-Chief

PLOS Pathogens

orcid.org/0000-0002-7699-2064
